# Pleistocene glacial refugia across the Appalachian Mountains and coastal plain in the millipede genus *Narceus*: Evidence from population genetic, phylogeographic, and paleoclimatic data

**DOI:** 10.1186/1471-2148-9-25

**Published:** 2009-01-30

**Authors:** Matt J Walker, Amy K Stockman, Paul E Marek, Jason E Bond

**Affiliations:** 1 East Carolina University, Department of Biology, Howell Science Complex N211, Greenville, NC 27858, USA

## Abstract

**Background:**

Species that are widespread throughout historically glaciated and currently non-glaciated areas provide excellent opportunities to investigate the role of Pleistocene climatic change on the distribution of North American biodiversity. Many studies indicate that northern animal populations exhibit low levels of genetic diversity over geographically widespread areas whereas southern populations exhibit relatively high levels. Recently, paleoclimatic data have been combined with niche-based distribution modeling to locate possible refugia during the Last Glacial Maximum. Using phylogeographic, population, and paleoclimatic data, we show that the distribution and mitochondrial data for the millipede genus *Narceus *are consistent with classical examples of Pleistocene refugia and subsequent post-glacial population expansion seen in other organismal groups.

**Results:**

The phylogeographic structure of *Narceus *reveals a complex evolutionary history with signatures of multiple refugia in southeastern North America followed by two major northern expansions. Evidence for refugial populations were found in the southern Appalachian Mountains and in the coastal plain. The northern expansions appear to have radiated from two separate refugia, one from the Gulf Coastal Plain area and the other from the mid-Atlantic coastal region. Distributional models of *Narceus *during the Last Glacial Maximum show a dramatic reduction from the current distribution, with suitable ecological zones concentrated along the Gulf and Atlantic coastal plain. We found a strong correlation between these zones of ecological suitability inferred from our paleo-model with levels of genetic diversity derived from phylogenetic and population estimates of genetic structuring.

**Conclusion:**

The signature of climatic change, during and after the Pleistocene, on the distribution of the millipede genus *Narceus *is evident in the genetic data presented. Niche-based historical distribution modeling strengthens the conclusions drawn from the genetic data and proves useful in identifying probable refugia. Such interdisciplinary biogeographic studies provide a comprehensive approach to understanding these processes that generate and maintain biodiversity as well as the framework necessary to explore questions regarding evolutionary diversification of taxa.

## Background

The profound effect of Pleistocene glaciation upon species distributions in the Northern Hemisphere has been well documented [1-7]. As continental ice sheets advanced into temperate areas, rendering once suitable areas uninhabitable, many northern taxa experienced extreme range reduction and fragmentation into refugia, while other populations and taxa were decimated entirely. Subsequently, as the ice sheets receded, recolonization of these historically glaciated areas by northernmost populations in refugia occurred rapidly. The genetic population structure in these organisms often reflects geological patterns [1,2,7]. Rapid, post-glacial expansions distribute internal (ancestral) haplotypes throughout large, previously glaciated, geographic areas. Furthermore, exponential growth of founding populations and subsequent resource competition may prevent other refugial populations from expanding simultaneously [4]. Taxa currently occupying previously glaciated and historically unsuitable areas are often characterized by low levels of genetic diversity [1-3,5-7].

Approximately 22,000 to 19,000 years before present, global temperatures were significantly lower than today and the volume of land-based ice was at its maximum [8]. Recently, niche-based species' distribution modeling has been applied to construct habitat suitability models for taxa during the Last Glacial Maximum [9-11]. Increasing availability of high-resolution temporal and spatial paleoclimatic data have greatly aided and improved fine-scale modeling of species' historical geographic movements. The number of studies showing the presence of genetic signatures indicative of glacial refugia among terrestrial and aquatic species has increased dramatically since the 1990's [4,10]. These models provide a relatively novel approach to exploring historical distributions of species, as they often coincide with previously hypothesized refugial regions [10].

**Figure 1:**
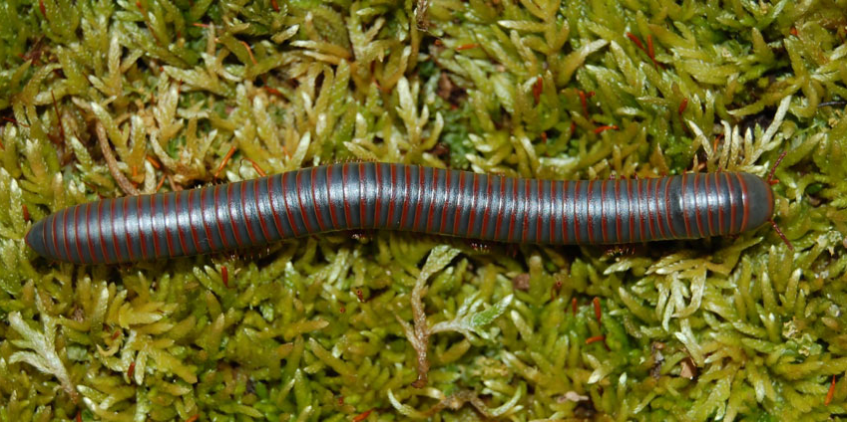
**Photograph of live *Narceus *specimen from upstate New York, USA**.

The millipede genus *Narceus *Rafinesque 1820 (Diplopoda, Spirobolida) is widely distributed throughout eastern North America with populations currently located in both historically glaciated and un-glaciated regions [12]. *Narceus *ranges from southern Quebec south through the Florida Keys and west to central Texas. *Narceus *specimens (Figure 1) are relatively large, cylindrical millipedes commonly found in Appalachian mixed mesophytic deciduous forests. They are primarily nocturnal, burrowing in rotten logs or leaf litter during the day and roaming about the forest floor or climbing trunks of large trees throughout the night. During the winter, they burrow deep into soil and re-emerge in the spring to feed on dead tree material and mate. *Narceus *are some of the most commonly encountered millipedes in the Appalachian Mountains (often referred to as *iron worms *by locals). They are often seen in large numbers just after rainfall or sometimes during the night. Whereas most millipedes are intolerant of dry, warm temperatures, *Narceus *species seem to have a wide range of moisture and heat tolerance and are often encountered crossing asphalt roads during the summer. Consequently their dispersal and colonization capabilities are likely greater than other co-distributed millipede groups (e.g., litter-dwellers like the orders Chordeumatida and Polydesmida).

With the exception of one morphologically distinct species in Florida, populations of *Narceus *appear phenotypically homogeneous throughout their range. However, their rather simplified morphology and current nomenclature belie a number of taxonomic complexities that have historically plagued this geographically widespread genus. At present, the genus comprises three species (*N. americanus*, *N. annularis*, and *N. gordanus*), which contain no fewer than 16 synonyms. Although resolving the taxonomy of this group is not our primary focus, the complex history of the genus (discussed herein) has likely contributed to confusing species boundaries within this ubiquitous and interesting group of millipedes. Given the history (evolution and nomenclatural) of the genus, as suggested by Shelley [13], molecular data are likely needed to *begin *to properly disentangle the species limits within *Narceus*. The union of phylogeographic and population genetic principles provides the toolkit necessary to investigate the historical distribution of these lineages and, ultimately, helps disclose the mechanisms that maintain current levels of diversity and population structuring.

Here we present the first phylogeographic study of the genus *Narceus*. We show that the genetic structure and current distribution of the genus *Narceus *(east of the Mississippi) reflects a complex history of refugia in the Appalachian Mountains and coastal plain of North America. We examine our data for patterns that should be consistent with a Pleistocene refugia hypothesis. First, there should be a profile of phylogeographic and genetic diversity that distinguishes coastal plain versus all other populations. Secondly, populations currently occupying historically unsuitable habitat should show genetic signatures of rapid range expansion. And last, high probability of habitat suitability from paleo-models of historical distributions should be coincident with those populations having relatively higher levels of genetic diversity.

## Results

### Summary of data and phylogenetic analyses

Mitochondrial sequence data (1354 bp) were obtained for individuals (n = 269) from 96 localities; 427 positions were parsimony informative (GenBank accession numbers FJ213618-FJ213764). The average ingroup sequence divergence was 7.3% with a range of < 0.01% – 14.6%. Most localities are represented by > 3 individuals. However at 10 localities we were only able to obtain a single individual. One hundred and fifty-three unique haplotypes were recovered following alignment. Twelve of these haplotypes were found at multiple localities, one of which occurred at 12 widely distributed northern localities. Of the 86 localities surveyed where more than a single individual was sampled (90%), 21 contained a single haplotype.

For the Bayesian analysis, GTR + I + Γ model of molecular evolution was inferred for the 16S region and HKY + I for both the tRNA-Val and 12S partitions. Of 8 million generations, 800,000 were discarded as burnin, resulting in a tree with a mean ln-likelihood value of -9768.09.

**Figure 2:**
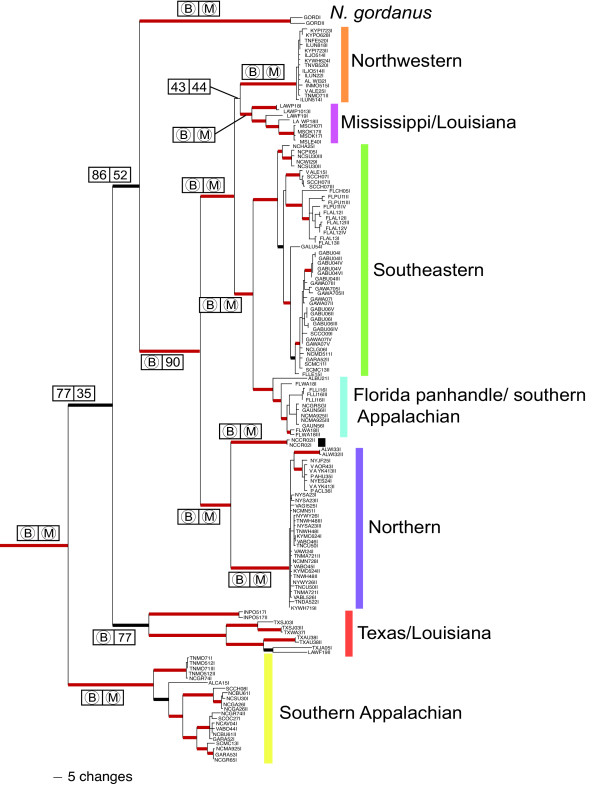
**Inferred mtDNA haplotype phylogeny for *Narceus *using Bayesian inference**. For major nodes, support values are represented in boxes. B and M represent posterior probabilities = 0.95 and maximum likelihood bootstrap values = 95%, respectively; PP and ML bootstrap values < 0.95 and 95% are represented by numbers in respective boxes. Thick red and thick black branches indicate PP = 1.00 and 0.70 – 0.95, respectively.

**Figure 3:**
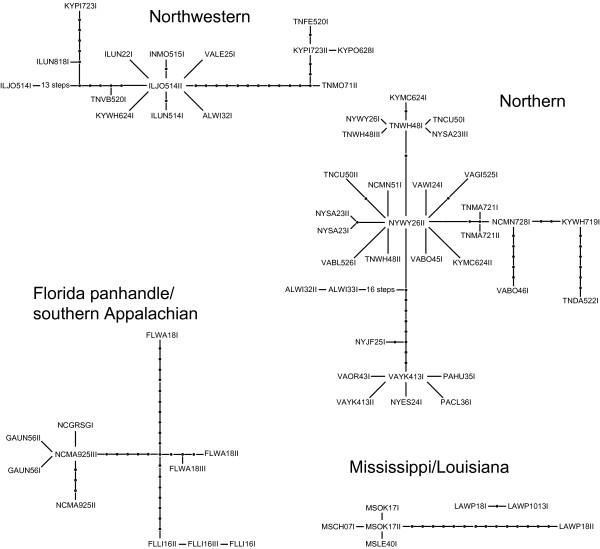
**mtDNA haplotype networks**. Haplotype networks recovered at the 90% confidence level using statistical parsimony. Clade labels correspond to those used in Figure 2.

**Figure 4:**
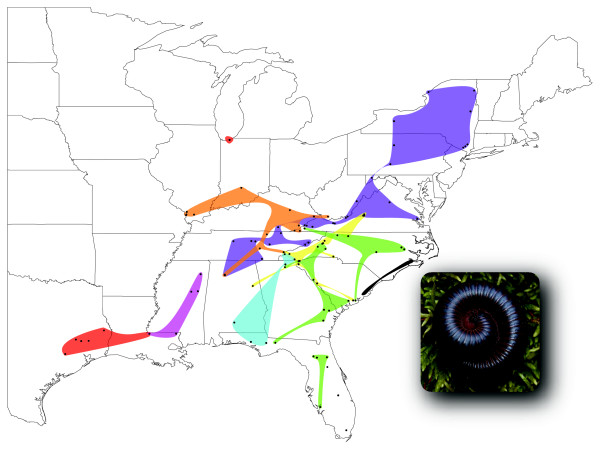
**Distribution of *Narceus***. Black circles represent localities of specimens collected. Colored areas correspond with colors used on the phylogenetic tree illustrated in Figure 2. Inset of *Narceus *specimen (not to scale; i.e., specimen much smaller, relative to land area represented in figure).

The Bayesian phylogeny (Figure 2) shows four distinct groups: 1) a Texas and Louisiana clade, 2) a Southern Appalachian clade, 3) a clade corresponding to the morphologically distinct *Narceus gordanus *(Chamberlin, 1943), and 4) a large, widely distributed sister clade to *N. gordanus *[14]. We used statistical parsimony at a 95% confidence level, implemented by TCS [15], to subdivide this larger clade. With a 95% reconnection limits most clades were connected, however, by relaxing the confidence level to 90% (a reasonable level given the relatively high ingroup sequence divergence) the networks were more inclusive and reflected the major clades recovered in the Bayesian tree with a higher degree of precision. Six networks (Figure 3) and two unconnected haplotypes were recovered, all of which agreed with the topology of the Bayesian phylogeny: a Northwestern clade, a Northern clade, two small Mississippi/Louisiana networks, a widespread Southeastern clade, a Florida panhandle/southern Appalachian clade, and two haplotypes that fall out on a single branch sister to the northern clade (Figures 2 &4). Most nodes are highly supported with posterior probabilities > 0.95 and, with the exception of two interior nodes and several very shallow nodes, all others are well supported (PP > 0.75). The haplotype networks of both northern clades reveal star-like patterns around each of the widespread haplotypes, a pattern indicative of the rapid expansion of ancestral haplotypes over a large geographic area [16,17].

### Phylogeography

**Figure 5:**
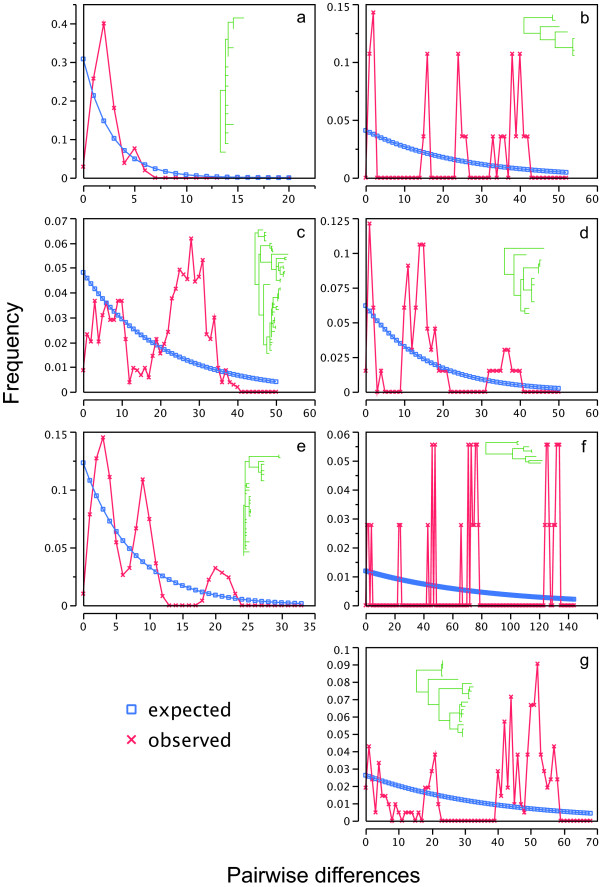
**Mismatch distribution plots for major *Narceus *clades**. Observed frequency of pairwise differences versus expected under exponential population growth model for major mtDNA lineages: (a) Northwest, (b) Mississippi/Louisiana, (c) Southeastern, (d) Florida panhandle/southern Appalachian, (e) Northern, (f) Texas/Louisiana, (g) Southern Appalachian. Corresponding clades are shown in the corner of each panel, illustrated in green.

Tests for isolation by distance were significant in all clades except the Northwestern clade (r = 0.0977, P = 0.2450) and a subclade of the Northern clade (r = 0.1345, P = 0.059). Although it is one of the more widespread clades, isolation by distance was not predicted in the Northwestern clade as it exhibits low levels of sequence divergence relative to the other clades in the phylogeny. Mismatch distribution plots (Figure 5) were multimodal in most cases, although the above-mentioned subclade of the Northern clade showed a smooth, unimodal curve. Fu's Fs was significant in the Northwestern clade (Fs = -12.15, P < 0.0001), Southeastern clade (Fs = -13.76, P < 0.0001), and the Northern clade (Fs = -20.20, P < 0.0001) and non-significant in all other clades (Mississippi/Louisiana Fs = -0.670, P = 0.225; Florida panhandle/southern Appalachian; Fs = -1.703, P = 0.177; Texas/Louisiana Fs = 0.650, P = 0.368; Southern Appalachian Fs = -0.539, P = 0.386).

**Figure 6:**
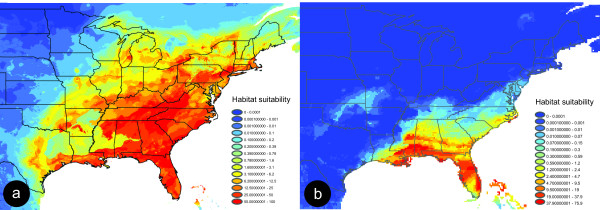
**Niche-based distribution model constructed in MAXENT using current and Pleistocene climatic parameters for *Narceus***. The models show the predicted distribution (habitat suitability) of *Narceus *during (a) the present and (b) the Last Glacial Maximum. Distribution models illustrate high levels of predicted habitat suitability in red and low levels in blue.

**Figure 7:**
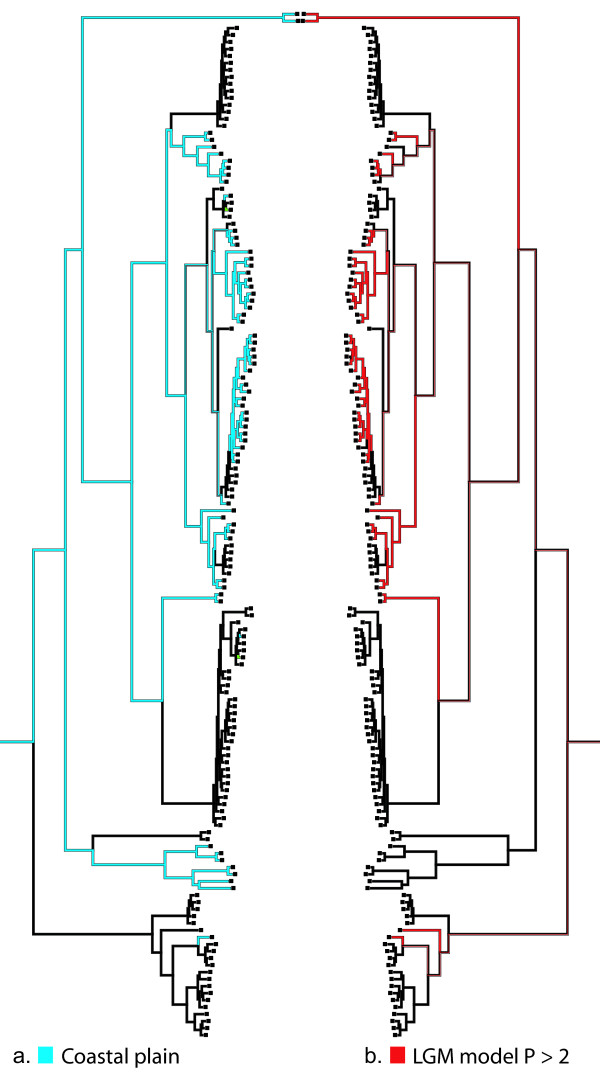
**Mirrored character state optimizations of coastal plain and paleo-presence threshold matrices onto the mtDNA haplotype phylogeny using a parsimony character reconstruction method**. (a) Optimization of the coastal plain (light blue branches) and "montane" (black branches) characters states. (b) Red branches indicate habitat suitability above the lowest point threshold (P = 0.2) derived from the niche-based paleodistribution model (values extracted from analysis illustrated in Figure 6b); black branches show optimization of character state that represents values where P < 0.2 (i.e., *Narceus *not expected to have occurred in these areas during the Pleistocene glaciation.

The coastal plain character optimization (Figures 6 and 7a) shows a complex phylogeographic pattern with nine independent colonizations into the Appalachian highlands and six re-colonizations back to the coastal plain.

### Niche-based distribution modeling

The western extent of present-day suitable habitat in the niche-based occurrence model (Figure 6a) is fairly consistent with data recovered from material deposited in museum collections [13]. Alternatively, the niche-based distribution model (Figure 6b) for *Narceus *during the Pleistocene shows a major reduction in habitat suitability during the Last Glacial Maximum. The highest probabilities of occurrence, representing ecological suitability during the Pleistocene, are found along the Gulf coast, throughout the Florida peninsula, and a minimal stretch of the Atlantic coast (Figure 6b). Tests for significant correlation between genetic diversity (Table 1; mean pairwise difference; π; *θ*; [18]) and regions of habitat suitability in the paleoclimatic distribution model were all significant (r_s _= 0.296, P < 0.001; r_s _= 0.308, P < 0.001; r_s _= 0.362, P < 0.001 respectively), showing a correlation between the observed high levels of genetic diversity and areas of Pleistocene refugia identified using the niche-based distribution model.

**Table 1:** Genetic divergence and diversity computed for each of the major *Narceus *clades; clade names correspond to those designated in Figure 2.

Clade	Mean pairwise distance	π Tajima, 1996	θ Tajima, 1996
Northern	0.00451	0.00178	0.00389
Northwestern	0.01792	0.00536	0.01010
Southeastern	0.02963	0.01515	0.02076
Florida panhandle/southern Appalachian	0.02909	0.01132	0.01512
Mississippi/Louisiana	0.03065	0.01754	0.01701
Texas/Louisiana/Indiana	0.10031	0.06223	0.05746
Southern Appalachian	0.04335	0.02848	0.02684

When we optimize the character "coastal plain" onto the haplotype phylogeny and then compare it to the optimization of suitable habitat during the Pleistocene (derived from the 0.2 threshold value in the MAXENT output) we find a very high level of similarity (Figure 7); we only recovered a single haplotype with a high probability of paleo-model habitat suitability not located in the coastal plain. A large majority of the coastal plain haplotype lineages occupy areas that would have been suitable refugia. Furthermore, each clade with a coastal plain interior node has a non-coastal plain sister clade. The Mississippi/Louisiana clade is sister to the exclusively non-coastal, shallowly divergent Northwestern clade; likewise, the Florida panhandle/Southern Appalachian clade is sister to the Southeastern clade, which comprises a non-coastal sister to a subclade showing a radiation from and back to the coastal plain. Genetic diversity for the coastal clade consisting of only two haplotypes (NCCR02II and NCCR02I) could not be calculated due to limited sampling. However, we do see a pattern similar to other sister clades within the tree.

## Discussion

Using an integrative approach that employs genetic and geospatial data, this study provides a straightforward example of Pleistocene refugia and post-glacial colonization. The star-like patterns and widely distributed haplotypes in our phylogeographic analyses taken together with significantly negative Fu's Fs values, support two major rapid expansions from coastal refugia into northern, historically glaciated areas. The southern populations of *Narceus *are characterized by relatively high levels of genetic diversity, isolation by distance, multimodal mismatch distributions, and an absence of widely distributed ancestral haplotypes. Similar genetic patterns across geographic regions attributed to Pleistocene related phenomena have been detected in numerous animals including salamanders [5,6], frogs [19], voles [20], mice [21], bears [3], and grasshoppers [22]. Our niche-based paleodistribution model also provides compelling evidence for southern refugia, specifically relegated to the coastal plain.

### Pleistocene refugia and post-glacial expansion

Glacial pulses during the Pleistocene profoundly affected animal distributions in North America, and species in the genus *Narceus *are no exception. As continental glaciers advanced, the destruction of habitat necessitated the displacement or extinction of northern taxa. Populations of displaced northern taxa suffered severe bottlenecks and hence decreased genetic diversity, while southern taxa that persisted during glacial advances were able to maintain high levels of diversity [4]. An example of this phenomenon was provided by Church *et al*. [5] in eastern tiger salamanders. They showed a distinct split between eastern and western populations. Within the eastern lineage, two possible refugia were identified: the Blue Ridge Mountains and the coastal plain of the Carolinas. They hypothesized that the populations located in mountain refugia have remained isolated after deglaciation whereas the coastal plain refugia were the source for northern post-glacial expansion along the east coast.

We found a similar pattern in *Narceus*. The basal-most clade in the phylogeny (Southern Appalachian) occurs south of any previously glaciated areas in the southern Appalachians, with populations from the Blue Ridge Mountains of Virginia to the southern extent of Appalachia in eastern Alabama. This clade also exhibits isolation by distance and relatively high levels of genetic diversity with no unique haplotypes occupying more than a single locality. The Texas/Louisiana clade, the next basal-most clade, also demonstrates the genetic signature of a refugial past. Within the Texas/Louisiana clade, mismatch distributions (Figure 5) indicate highly fragmented populations [23], with each locality sampled having at least one unique haplotype – another common characteristic of refugia populations [24,25]. With our sampling scheme, we found a single distributional extension into northern Indiana (Figures 2 &4), at first believed to be an anthropogenic introduction. However, we did detect isolation by distance within this clade. Without further sampling, the phylogenetic placement of this population remains enigmatic. A similar pattern of radiation was detected between the Mississippi/Louisiana clade and its sister, the Northwestern clade (Figures 2 &4).

The Mississippi/Louisiana clade exhibits isolation by distance, relatively high levels of genetic diversity, and a lack of shared haplotypes among localities – all characteristics consistent with a deep refugial history [4,6]. Its sister, the Northwestern clade, is widely distributed west of the Appalachians (Figure 4). This clade exhibits relatively low levels of genetic divergence and is distributed across a large geographic distance. It also contains a single haplotype (ILOJ514II) found in eight localities in four states (Illinois, West Virginia, Virginia, and Tennessee). The haplotype network indicates six other haplotypes connected to ILOJ514II by = 2 mutational steps, resulting in a star-like pattern (Figure 3). A pattern also observed for rapidly expanded populations of spotted salamanders [6], wood frogs [19], and other taxa [17,21,26-29]. The star-like pattern and the mismatch plot of pairwise differences in the Northwestern clade (Figure 5) are both consistent with relatively rapid expansion, as might be expected for a quick founding colonization into a newly deglaciated suitable area. Indeed, the results of the paleodistribution models (Figure 6) show that the majority of habitat currently occupied by the Northwestern clade was likely unsuitable 21,000 years ago. This pattern reflects a northern radiation from the Gulf coast northward and west of the Appalachians, resembling that of the Texas/Louisiana clade.

Similar to patterns found in eastern tiger salamanders [5], we detected a radiation from the mid-Atlantic coastal plain north through upstate New York; however, this radiation appears to have extended west as well, like that seen in the spotted salamander (Figure 4) [6]. The Northern clade has three widely distributed haplotypes all of which show star-like TCS networks (Figure 3). Fu's Fs, an indicator of rapid range expansion [30,31], was significant and negative for the Northern clade. Despite multiple lines of evidence indicating rapid range expansion, isolation by distance was detected for this clade. Although long-distance dispersal can also account for patterns similar to those seen for this clade, millipedes are non-vagile and thus unlikely to disperse, via a single event, across a long distance. Therefore, the phylogeographic pattern is more likely the result of rapid range expansion.

The Southeastern and Florida panhandle/southern Appalachian clades represent separate refugial populations that have remained isolated. The Southeastern clade indicates a rather complex phylogeographic pattern in which mismatch plots and isolation by distance analyses were consistent with those of refugial populations. However, Fu's Fs was significant and negative in the Southeastern clade, suggesting a rapid expansion. This clade is widespread throughout the southeast, and character map reconstructions suggest multiple radiations to and from the coastal plain within this clade. These pulses may have been fairly recent, a scenario consistent with significant negative values for Fs obtained here. The Florida panhandle/southern Appalachian area represents a coastal refugium with an east-to-west phylogeographic break at the Apalachicola/Chattahoochee/Flint (ACF) River Basin, a well documented geographic barrier to gene flow for multiple taxa [32]. There is a single radiation of these millipedes forming a monophyletic group extending southeast of the ACF barrier into the southern Appalachians. The Fall Line (the interface between the coastal plain and the Appalachian foothills) does not seem to prevent radiations of these millipedes; the area immediately east and running north-to-south of the ACF basin may serve as a riparian corridor from the coastal plain to the southern Appalachians [33].

### Paleodistribution modeling

High-resolution models of past distributions for species provide a promising new tool in the field of phylogeography [9,10]. A recent study by Waltari *et al*. [10] addressed concordance between Pleistocene refugia predicted using traditional biogeographic methods and niche-based paleodistribution modeling. Fourteen of the twenty North American terrestrial taxa tested showed significant spatial correlations between traditional and niche-based modeling methods for identifying Pleistocene refugia [10]. Agreement between these methods suggests that paleodistribution models may well provide useful guidelines for biogeographic studies.

Our Last Glacial Maximum model for *Narceus *(Figure 6b) indicates a major reduction in habitat suitability that stands in stark contrast to the present-day model. Hypotheses of refugia along the Gulf and Atlantic coastal plains are consistent with indicated areas of highest predicted suitability in the niche-based distribution models. The character map of haplotypes by region, illustrates a disproportionate number of haplotypes from these areas on the phylogeny (Figure 7). A correlation between genetic diversity and habitat suitability from our Pleistocene model suggests that the model's highest predicted probabilities, in fact, do show higher levels of genetic diversity and are consistent with those patterns expected from refugial areas. These refugia hypotheses are consistent with all mismatch plots and isolation by distance tests for clades occurring in coastal areas.

Somewhat inconsistent with the refugia hypotheses was the low habitat suitability modeled in the southern Appalachian and Texas coastal plain regions of our paleodistribution model. Although these areas show very low probability (0.007% – 1.390%) of having suitable habitat during the Last Glacial Maximum (Figure 6), it is apparent that millipede populations were able to survive there. During the Last Glacial Maximum, suitable habitat in these areas may have been reduced into numerous microrefugia within the overall southern Appalachian refugium that could lead to the diversity currently found in these clades. Under this hypothesis, overall habitat suitability would appear to be very low; however, populations may have persisted within suitable microrefugia.

### Taxonomic considerations

As discussed in the Background section, *Narceus *presently comprises three nominal species, two of which appear to be morphologically homogenous. *Narceus gordanus *is the only species that appears to be morphologically distinct and has a rather limited distribution, endemic to peninsular Florida and extending north to South Carolina [34]. Individuals assigned to *N. gordanus *are represented in the phylogeny (Figure 2) by a clade nested squarely within populations that are morphologically indistinguishable and clades that likely represent the remaining two species. This phylogenetic placement renders *N. americanus *and *N. annularis *(*sensu lato*) paraphyletic. Moreover, as has been demonstrated for other millipede groups [e.g., Jamaican *Anadenobolus*; [35,36]], cryptic species may be a problem for species delineation within this genus. Further work will be needed to resolve these issues, both in terms of careful morphological analyses and intensive collecting west of the Mississippi. An integrative approach that takes into account biogeographic patterns, analyses of morphology, phylogenetic history, and climatic envelope will likely provide the framework necessary to investigate species boundaries in this widespread, morphologically homogeneous genus [see [37]].

## Conclusion

The signature of climatic change on the distribution of the millipede genus *Narceus *is evident in the mtDNA data presented here. Niche-based historical distribution modeling strengthens the conclusions drawn from the genetic data and proves useful in identifying areas of probable Pleistocene refugia. This study provides the first molecular phylogeographic and ecological modeling data for the millipede genus *Narceus*. The methods used here provide an integrative biogeographic approach to understand processes generating patterns of biodiversity, and an approach to explore the evolutionary diversification of species, populations, and organismal distributions.

## Methods

### Molecular Protocols and sequence alignment

Live millipedes collected in the field were transported to the laboratory, where legs or heads were removed and stored in RNA *later *(Qiagen, Valencia, CA) at -80°C. Voucher specimens were stored in 80% ethanol and will be eventually deposited in the Field Museum of Natural History collection (Chicago, IL). Genomic DNA was extracted from leg or head tissue using a Qiagen DNeasy Tissue Kit (Qiagen, Valencia, CA) and a mitochondrial region spanning 12S, tRNA-Val, and 16S genes was amplified via a standard polymerase chain reaction (PCR) with primers LR-J-12887dip (CCG GTC TGA AAC TCA GAT CAT GT) and SR-N-145xxdip1 (GTA TAT CGC TGT CGT CAG A). Products were column purified and sequenced with an ABI Hitachi 3130 Genetic Analyzer and/or an ABI PRISM 377 automated DNA sequencer. Raw sequence data were edited with Sequencher 4.1. Multiple sequence alignment was performed with MUSCLE [38]. Aligned data were partitioned by comparison with a published mitochondrial genome of *Narceus *[39].

### Phylogenetic Analyses

One hundred and fifty three unique haplotypes (1354 bp) were used as terminal taxa for phylogenetic analyses. The molecular data were divided into 3 partitions: 16S, tRNA-Valine, and 12S. Models of nucleotide evolution for each partition were selected using MRMODELTEST 2.2 [40] following the Akaike Information Criterion (AIC). Phylogenetic reconstructions were created using both maximum likelihood in GARLI 0.95 (1000 bootstrap replicates; program available at ) and Bayesian inference in MRBAYES 3.1.2 [41]. With MRBAYES, two simultaneous analyses were run, each with eight chains sampling every 100 generations. Convergence was assumed as the average standard deviation of split frequencies stabilized below 0.01. TRACER 1.4 (available at ) was employed to examine parameter values, and a majority-rule consensus tree was produced after the removal of trees saved prior to burn-in. Statistical parsimony, as implemented by TCS [15], was used as an unbiased criterion for assigning subclades.

### Phylogeographic

Isolation by distance was tested within each major mtDNA lineage using a Mantel test of correlation between genetic and geographic distance matrices implemented in the software package IBD [42]. Using DNASP [43], mismatch distributions of pairwise differences within each major mtDNA lineage were produced and plotted against the expected distributions under a null model of exponential population growth to explore the possibility of recent range expansions [23]. Unimodal distributions are expected for clades with recent population expansions, whereas multimodal distributions are expected for clades with more phylogeographic structure. In terms of refugia scenarios, an unimodal distribution of pairwise differences would be expected for shallowly divergent lineages that currently occupy previously glaciated areas, and multimodal distributions would be expected for deeply divergent lineages that currently occupy areas once suitable for glacial refugia [28]. We employed DNASP to compute Fu's Fs (1000 replicates), shown in a comparative study by [30] to be one of the best indicators of rapid population growth.

Phylogenetic patterns for coastal plain populations were investigated by scoring haplotypes as coastal or "montane" and then using the parsimony character state reconstruction in MESQUITE [44] to optimize these two character states onto the haplotype phylogeny. As a direct confirmation to assess whether coastal plain regions may have served as refugia for *Narceus *during the glacial maximum, values were extracted from the niche-based distribution model using the paleoclimatic data set (see methods below). We scored two character states: 0) area suitable during the Pleistocene (P ≥ 0.20); and 1) area unsuitable during the Pleistocene (P < 0.20).

### Distribution modeling

Niche-based distribution modeling explores algorithmic approaches to extracting associations between geographical occurrence of species and a given set of environmental variables. Once the relationships between species occurrences and environmental variables has been established, predictions of the focal species' distribution and locations of potentially suitable habitat can be generated [45,46]. Although most commonly employed to predict the current distribution of a species, distribution models can also be projected onto hypothesized past and future environments to reconstruct a species distribution at any point in time and space [9,11,47,48]. To compare the current distribution of *Narceus *with its predicted distribution during the Last Glacial Maximum, we employed the program MAXENT [45]. MAXENT is a machine-learning method that estimates the least biased focal probability distribution by maximizing the distribution's entropy within the constraints of the known data [45]. There are a variety of algorithms and computer programs available for niche-based distribution modeling, but several studies have shown MAXENT to be one of the more robust [45,49,50]. For our environmental data layers, we began with 19 standard temperature and precipitation variables based on interpolated climate data at a resolution of 30 arc-seconds [WorldClim; [51]]. The values of each variable for each specimen locality were extracted and analyzed for correlation. Highly correlated variables (r-squared >= .85) were subsequently removed resulting in ten non-redundant [52] environmental variables (Table 2). Paleodistribution models were based on a matching set of environmental data layers for the Last Glacial Maximum. The paleoclimatic layers for 21,000 years before present were generated and provided by Richards *et. al*. [9] using the Community Climate Model [53] which incorporates atmospheric dynamics, including radiative and convective processes, condensation, and evaporation.

**Table 2:** Environmental variables used to produce niche-based distribution models.

Environmental variables
Annual mean temperature
Temperature seasonality (standard deviation *100)
Mean temperature of wettest quarter
Mean temperature of driest quarter
Mean temperature of warmest quarter
Mean temperature of coldest quarter
Annual precipitation
Precipitation seasonality (Coefficient of variation)
Precipitation of wettest quarter
Precipitation of driest quarter

We tested for significant correlations (Spearman's rho) between the Pleistocene habitat suitability and genetic diversity (mean pairwise difference; π; *θ*) [18] among major mtDNA lineages. Given that we have produced an accurate model and the fact the *Narceus *have limited dispersal capabilities, we would expect to see higher levels of genetic diversity in lineages currently occupying areas of high habitat suitability in our Pleistocene distribution model. Because MAXENT output is continuous, a threshold must be selected in order to create a binary distribution model where all pixels are either predicted as present or absent (or suitable/unsuitable). We chose a conservative threshold based on the lowest predicted value associated with any of the observed sampling sites for the current model [Pearson *et. al*.'s 'LPT'; [50]]. This approach maintains a zero omission error (i.e., zero false negatives), thereby maintaining that each pixel predicted "present" has conditions at least as suitable as those where the species is known to occur [50]. This threshold was then applied to the paleodistribution model and used as the basis of a character matrix. Thus, each haplotype in the inferred Bayesian phylogeny was marked as being located in either suitable or unsuitable habitat during the Last Glacial Maximum. Character maps were then reconstructed using parsimony in MESQUITE [44] to infer species movement and/or stasis between the past and present. Haplotypes located in areas predicted as suitable during the Last Glacial Maximum outline possible refugia areas, while those in locations predicted as unsuitable might be indicative of post-glacial range expansions.

## Authors' contributions

MJW conducted the fieldwork, carried out the molecular genetic studies and analyses, and drafted the manuscript. AKS participated in the design of the study, the niche-based distribution modeling, and reluctantly submitted the sequences to GenBank. PEM participated in the fieldwork, data collection and analysis. JEB conceived of the project, directed its design and coordination, and assisted in constructing and writing the paper. All authors assisted in the drafting of the manuscript and have read and approved the final version.
